# Real-life prevalence of resistance-associated variants against non-structural protein 5A inhibitors and efficiency of Daclatasvir + Asunaprevir therapy in Korean patients with genotype 1b hepatitis C

**DOI:** 10.1186/s12985-017-0826-1

**Published:** 2017-08-24

**Authors:** Jung Hwan Yu, Jung Il Lee, Kwan Sik Lee, Ja Kyung Kim

**Affiliations:** Gangnam Severance Hospital, Department of Internal Medicine, Yonsei University College of Medicine, 20, 63-gil, Eonju-ro Gangnam-gu, Seoul, 06229 South Korea

**Keywords:** Direct-acting antivirals, Chronic hepatitis C, Daclatasvir, Asunaprevir

## Abstract

**Background:**

Direct-acting antivirals (DAAs) for chronic hepatitis C (CHC) treatment are tolerable and highly effective in a shorter period of time than before. However, resistance-associated variants (RAVs) can affect the efficacy of DAAs. The aim of this study was to investigate the real-life prevalence of RAVs against non-structural protein 5A (NS5A) inhibitors in Korean patients with genotype 1b chronic hepatitis C.

**Methods:**

All consecutive patients with CHC genotype 1b who underwent a RAV test at a single referral hospital were enrolled.

**Results:**

A total of 142 patients (male 53, female 89) were tested for RAVs. The average age of the patients was 58 years. Liver cirrhosis was found in 34.5% (49/142) of patients, and 19.0% (29/142) of patients had previously undergone interferon-based treatment. Twenty-nine patients (20.4%) had RAVs (Y93 or L31). Y93H, L31, or Y93H with L31 were detected in 22 (15.5%), 8 (5.6%), and 1 (0.7%) patients, respectively. The presence of RAV was not affected by previous interferon-based treatment or by the existence of liver cirrhosis. Among 113 patients without baseline NS5A RAVs, 72 patients started daclatasvir (DCV) + asunaprevir (ASV) treatment and 95% (68/72) patients achieved virologic response at week 4. Virologic response at end of treatment and sustained virologic response at 12 weeks after treatment were achieved by 94% (68/72) and 94% (68/72), respectively.

**Conclusions:**

In Korean patients with genotype 1b CHC, 20.4% (29 of 142) of patients showed RAVs against NS5A inhibitors. Patient without RAVs who received treatment with DCV + ASV showed high virologic response rates in Korea.

## Background

Chronic hepatitis C virus (HCV) infection is one of the main causes of liver cirrhosis and hepatocellular carcinoma and is a principal cause of liver-related morbidity and mortality worldwide. Previously, the standard treatment for patients with chronic hepatitis C (CHC) included pegylated-interferon alpha (PEG-IFNα) and ribavirin therapy. [1] Treatment response was not only related to HCV genotype and the host’s interleukin (IL) 28B polymorphism but also related to the tolerability of the drugs. Although Korean patients have favorable single nucleotide polymorphisms near the *IL28B* gene, such as rs12979860CC and rs8099917TT allele, [2] poor tolerability has led to the hesitation to use interferon (IFN)-based treatment.

Recently, direct-acting antivirals (DAAs)—have been developed and substituted IFN-based regimen to treat patients with CHC. These DAAs are substantially more tolerable and effective than PEG-IFNα and ribavirin. [3–6] DAAs are molecules that target specific nonstructural (NS) proteins of the virus and results in disruption of viral replication and infection. There are four classes of DAAs, which are defined by their mechanism of action and therapeutic target. The four classes are NS proteins 3/4A protease inhibitors, NS5B nucleoside polymerase inhibitors, NS5B non-nucleoside polymerase inhibitors, and NS5A inhibitors. [7] Among the DAAs, combination treatment with daclatasvir (DCV) of NS5A inhibitor and asunaprevir (ASV) of NS3 protease inhibitor was introduced using multiple classes of DAAs with non-overlapping targets. These regimens showed a good treatment outcome in clinical trials of patients with CHC genotype 1b, regardless of IFN-intolerance or lack of response to IFN-based regimens. [8–10] Based on its efficacy and safety compared to that of IFN-based therapy, DCV + ASV combination therapy was the first IFN-free regimen reimbursed by national health insurance in Korea for the treatment of genotype 1b CHC.

However, a NS5A inhibitor, such as daclatasvir, has limited efficacy with baseline resistance-associated variants (RAVs) at NS5A-Y93H and NS5A-L3l. DCV + ASV combination therapy also showed various results depending on the presence of RAVs. According to a previous study, in patients with a sustained virologic response at 24 weeks post-treatment, the virus was eliminated in 98.6% of patients without NS5A polymorphism and in 42.1% of patients with NS5A polymorphism. [11] Thus, regarding the efficacy of DCV + ASV therapy, the presence of RAVs, especially the presence of NS5A RAVs, can be an important factor. However, impact of RAVs is regimen specific, since reports have shown that SVR rates after DCV combined with a different DAA was not influenced from NS5A RAVs. [12, 13]

NS5A RAVs prevalence varied from 18% (population-based sequencing) [6] to 29% (deep sequencing) in Japanese patients. [14] As the prevalence of HCV genotypes is quite different depending on the region, NS5A RAVs can vary depending on the region or the country in which it is treated, and the results and effects of DCV + ASV therapy are assumed to vary accordingly. Thus, when using DAAs, including NS5A inhibitors, investigating the real-life prevalence of NS5A RAVs in a specific area and its influence is important. The aim of this study was to investigate the real-life prevalence of RAVs against NS5A inhibitors in Korean patients with genotype 1b CHC and the efficiency of the treatment with DCV + ASV in patients with genotype 1b CHC without RAVs.

## Methods

### Patients

All consecutive patients with CHC who took the NS5A RAVs test from August 2015 to May 2016 were enrolled. Medical records were retrospectively reviewed, and data were collected from a single referral hospital, in Seoul, Korea. Patients were at least 20 years of age, with confirmed CHC genotype 1b infection and HCV RNA levels ≥10,000 IU/ml. Liver cirrhosis (LC) was diagnosed clinically by morphologic changes of cirrhosis on imaging studies or other signs of portal hypertension, such as portosystemic shunt or hypersplenism. This study was approved by the ethics committee of our hospital, and the need for informed consent was waived.

### Laboratory tests

HCV RNA was quantified using the Roche COBAS TaqMan assay (Roche Molecular Diagnostics, Pleasanton, CA, USA) with a lower limit of quantification of 15 IU/mL. HCV genotype and subtype were assessed using HCV genotyping kit (Biosewoom Inc., Seoul, Korea). The sequencing of a 408 bp fragment in the core gene and 293 bp fragment in the 5′ untranslated region (UTR) were used to assign genotypes. Genotypes of the strains were analyzed using the HCV sequence database (https://hcv.lanl.gov).

Direct sequencing of HCV NS5A Y93 and L31 gene regions from plasma samples was performed. Viral RNA was isolated from 200 μL plasma samples utilizing the QIA Amp MiniElute Virus Vacuum Kit (Catalog No. 57714 Qiagen, Inc. Valencia, CA) and the QiaCube workstation. Viral RNA was eluted in 25 uL of elution buffer and 20 uL of eluate was used in the cDNA preparation. The extracted RNA was reverse transcribed and amplified by the PCR method using the SuperScript III One-Step RT-PCR System with Platinum Taq DNA Polymerase (Invitrogen) with the pairs of primers as follows: sense (5872–5891) 5′-AAGAGGCTCCACCAGTGGAT-3′ and antisense (6730–6749) 5′-CGCCGGAGCGTACCTGTGCA-3′. The PCR products were purified using a QIAquick PCR Purification Kit (QIAGEN) and sequenced using an automated DNA sequencer (3730xl DNA Analyzer, Applied Biosystems). Two primer sets for sequencing through the NS5A L31 and Y93 coding regions are: forward sequencing primer (1bseqF2) 5′-TCCGGCTCGTGGCTAAGAGATGTTTGGG-3′ and reverse sequencing primer (1bseqR5) 5′-CAGTGGTCATGCCCGTCACGTAGTG-3′. Each sequence was confirmed for the sense and antisense strands. If minor sequences of RAV were detected in more than 10% of the strength of the major sequence, it was regarded as RAV positive. [15]

### Treatment

HCV treatment was based on guidelines of the Korean Association for the Study of the Liver (KASL). [16] According to the guidelines presented in 2015, patients with NS5A RAVs were recommended to be treated with ledipasvir/sofosbuvir. If patients without NS5A RAVs agreed to CHC treatment with firstly approved DAAs, DCV + ASV, they received DCV (one 60 mg tablet once daily) and ASV (one 100 mg capsule twice daily) combination therapy for 24 weeks as guidelines recommended. Patients were followed-up during treatment and for more than 12 weeks. Virologic response was defined as undetectable HCV RNA level at each time point. Virologic responses on week 4 of treatment, at the end of treatment, and 12 weeks post-treatment were checked. Before starting these therapies, the presence of current liver cirrhosis or a past patient’s history of IFN-based treatment was not considered for exclusion. However, patients who were currently being treated for HCC or refused to be treated were excluded from receiving DCV + ASV therapy.

### Statistical analysis

Continuous variables were presented as mean ± SD or median (interquartile range). Categorical variables were compared between groups by the chi-squared test. The Student’s t-test was used to compare the mean of continuous variables. A value of *p* < 0.05 was considered statistically significant. All statistical tests were performed with PASW version 17.0 (IBM Corp., Armonk, NY, USA).

## Results

### Baseline characteristics of patients

A total of 142 patients (male 53, female 89) were tested for RAVs at NS5A-Y93 and NS5A-L31. The average age of the patients was 58 years old (range: 24–90). LC was found in 34.5% (49/142) of patients, and 19.0% (27/142) of patients had previously undergone IFN-based treatment. Among the patients who underwent IFN-based treatment, 22 failed the treatment and 5 discontinued the treatment because of adverse hematologic events. Among the patients without NS5A RAVs, 72 patients underwent DCV + ASV therapy for 24 weeks. A comparison of the characteristics of patients in the RAV-positive and RAV-negative groups is shown in Table 1. RAV-positive patients were significantly older than RAV-negative patients. Other factors were not different between the groups.

**Table 1:** Baseline characteristics and laboratory findings of patients who underwent RAV test

	Total (*n* = 142)	RAV negative (*n* = 113)	RAV positive (*n* = 29)	*P* value
Male, n (%)	53 (37.3%)	43 (38%)	10 (35%)	0.723
Age, years	58 ± 14	55 ± 14	66 ± 11	< 0.001
LC, n (%)	49 (34.5%)	36 (32%)	13 (45%)	0.190
Treatment experienced, n (%)	25 (17.6%)	19 (17%)	6 (21%)	0.624
AST, IU/L	58.4 ± 36.5	59.1 ± 38.9	56.0 ± 25.9	0.685
ALT, IU/L	51.5 ± 52.0	52.9 ± 55.5	46.0 ± 35.3	0.526
Total bilirubin, mg/dL	0.91 ± 0.52	0.92 ± 0.5	0.87 ± 0.34	0.601
HCV RNA, × 10^6^ IU/mL	2.241 ± 2.996	2.046 ± 3.024	3.013 ± 2.799	0.127

All results are presented as n (%) or mean ± SD

*LC* liver cirrhosis, *AST* aspartate aminotransferase, *ALT* alanine aminotransferase, *RAV* resistance-associated variant

### Real-life prevalence of NS5A RAV

**Fig. 1:**
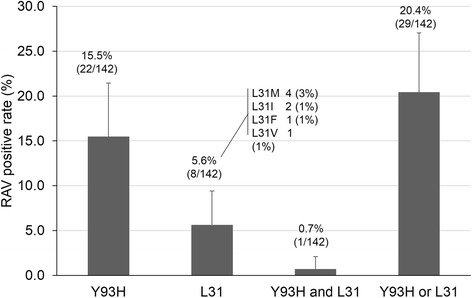
Real-life prevalence of NS5A resistance-associated variants (RAVs) in patients with chronic hepatitis C genotype Ib

**Fig. 2:**
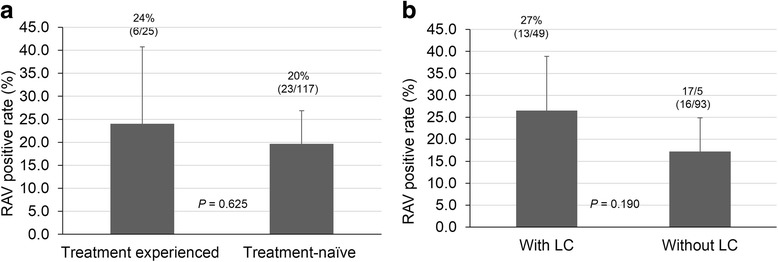
Comparison of non-structural protein 5A resistance-associated variants (RAVs) prevalence based on previous interferon-based treatment experience (**a**) and liver cirrhosis (LC) (**b**) in patients with chronic hepatitis C genotype 1b

Of the 142 patients, 22 patients (15.5%) had a Y93H RAV, and 8 patients (5.6%) had an L31 RAV in the NS5A RAV test. When considering L31 RAV, L31 M was the most common, being present in four patients, followed by L31I, present in two patients, and L31F and L31 V, which were present in one patient each. Moreover, one case had both Y93H and L31 V RAVs. Hence, 29 of the 142 patients (20.4%) had at least one NS5A RAV (Fig. 1). When comparing the RAV-positive patients who had undergone IFN-based treatment with treatment-naïve patients, 24% (6/25) of the patients who had undergone IFN-based treatment had RAVs, and 20% (23/117) of the treatment-naïve patients had RAVs. Furthermore, 27% (13/49) of patients who had cirrhosis had RAVs, while only 17% of the patients (16/93) without cirrhosis had RAVs (Fig. 2).

### Efficacy of DCV + ASV combination therapy

**Fig. 3:**
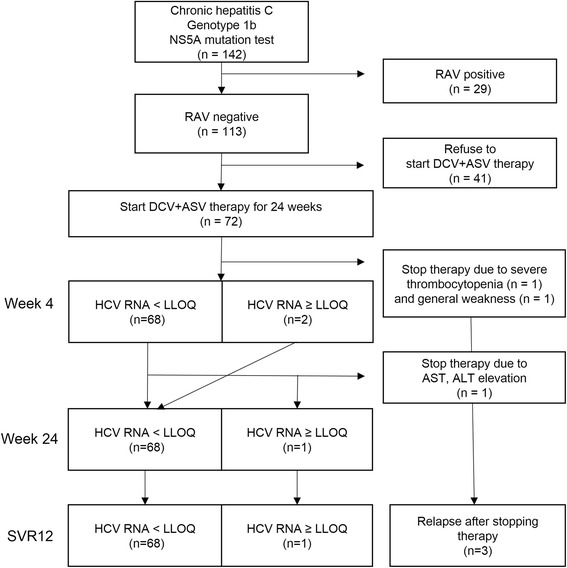
Flow chart of the participants and the outline of study. Among resistance-associated variants (RAVs) negative patients, 72 patients who agreed to start daclatasvir plus asunaprevir (DCV + ASV) therapy were analyzed for treatment response. Virologic response was determined if a level of serum HCV RNA is less than lower limit of quantification (LLOQ)

**Fig. 4:**
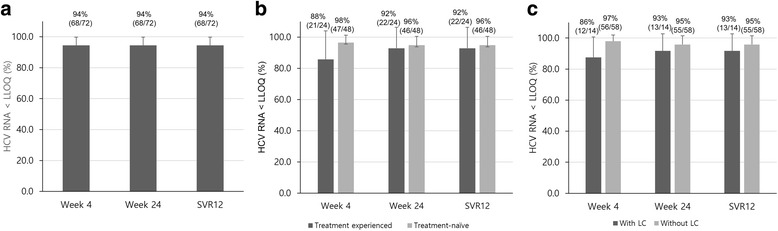
Efficacy of daclatasvir plus asunaprevir combination therapy for chronic hepatitis C genotype 1b: virologic response in total patients (**a**), comparison of virologic response based on liver cirrhosis (LC, **b**) and previous treatment experience (**c**)

A total of 72 of 113 RAV-negative patients started DCV + ASV therapy. Two patients stopped DCV + ASV medication because of adverse events within four weeks of starting the treatment. Other 2 patients who had low detectable levels of HCV RNA at week 4 continued the medication, and finally achieved virologic response at week 24 and SVR12. Among the 68 patients who achieved virologic response at week 4, only 1 patient did not achieve virologic response at week 24. Furthermore, 1 patient who achieved virologic response at week 4 stopped medication because of liver enzyme elevation at week 7 without virologic rebound during the therapy (Fig. 3). This patient experienced virologic rebound only after stopping medication. In summary, 94% (68/72) patients achieved virologic response at week 4. Virologic response at week 24 and SVR12 were achieved by 94% (68/72) and 94% (68/72) of patients, respectively (Fig. 4a).

Treatment-naïve patients showed virologic response rates at week 4, week 24 and SVR12 as 97%, 95% and 95%, respectively. Patients who had received IFN-based treatment previously showed virologic response rates at week 4, week 24 and SVR12 as 86%, 93% and 93%, respectively. However, no significant differences were shown by experience of IFN-based treatment (Fig. 4b).

Comparing the responses depending on the presence of cirrhosis, 95% (55/58) of patients who did not have accompanying cirrhosis achieved SVR12, and 93% (13/14) of patients who had cirrhosis achieved SVR12. There was little difference between the patients with and without accompanying cirrhosis in terms of achieving virologic response at week 4 and 24 (Fig. 4c).

### Characteristics of patients who discontinued DAA treatment

After the start of DCV + ASV treatment, 3 patients finally discontinued treatment because of adverse events (Table 2). One patient who had underlying idiopathic thrombocytopenic purpura (ITP) was confirmed to have AST/ALT elevation at 7th week of treatment. In this case, the patient had no choice but to stop the therapy because her AST/ALT levels increased to greater than 10 times of normal limit. In the second case, the patient was diagnosed with petechia, which occurred in the whole body, from the second week of the treatment. Because the patient had a history of cured ITP 20 years ago, an initial impression of recurred ITP was suspected in the emergency room blood test.Since no causes other than the newly administered DCV + ASV were found, the patient stopped taking DCV + ASV therapy, and the platelet count recovered after a steroid treatment. The patient is now under follow-up care after hospital discharge. In the last case, the patient wanted to stop treatment because of severe general weakness and fatigue at the second week of treatment. All these patient had viral rebound viral after halting treatment.

**Table 2:** Patients’ characteristics who discontinued daclatasvir plus asunaprevir treatment

	Reference levels	Patients who discontinued treatment because of adverse events		
		Patient 1	Patient 2	Patient 3
Cause of discontinuation		AST/ALT elevation	Thrombocytopenia	General weakness Fatigue
Toxicity grade by CTCAE		Grade 3	Grade 4	Grade 3
Age/Sex		45/F	49/F	79/F
HCV RNA, IU/ml		194,000	1,390,000	5,620,000
Liver cirrhosis		No	No	Yes
Comorbidities		ITP	HTN	HTN, DM
Baseline				
White blood cells, ×10^9^/L	4.0–10.8	6150	6800	7290
Hemoglobin, g/dL	12–15.9	12.6	13.9	16.5
Platelets,×10^9^/L	150–400	23	158	144
AST/ALT, IU/L	13–36/1–46	21/27	49/54	88/97
Total bilirubin, mg/dL	0.2–1.3	0.5	0.7	0.9
Albumin, mg/dL	3.4–5.3	3.6	4.1	3.9
Prothrombin time, INR	0.96–1.16	1.21	1.04	1.17
At week 4				
White blood cells, ×10^9^/L	4.0–10.8	6410	7990	10,770
Hemoglobin, g/dL	12–15.9	14.0	13.4	17
Platelets, ×10^9^/L	150–400	19	< 5	160
AST/ALT, IU/L	13–36/1–46	265/450	29/15	86/101
Total bilirubin, mg/dL	0.2–1.3	1.0	0.6	1.0
Albumin, mg/dL	3.4–5.3	4.1	4.3	4.4
Prothrombin time, INR	0.96–1.16	1.41	0.99	1.13

*CTCAE* common terminology criteria for adverse events, *ITP* idiopathic thrombocytopenic purpura, *DM* diabetes mellitus, *HTN* hypertension, *AST* aspartate aminotransferase, *ALT* alanine aminotransferase, *INR* international normalized ratio

## Discussion

Development of new drugs for HCV infection, one of the world’s major health crises, provides an opportunity to come one step closer to its complete cure. In particular, it provides an opportunity for complete cures for patients who have failed IFN and ribavirin treatment, thus allowing easier treatment because of fewer adverse effects. DAAs mainly act on nonstructural proteins that are formed by the HCV. RAVs are known to drastically reduce the efficiency of these drugs [6, 17, 18]. Therefore, when prescribing these drugs, such as DCV + ASV, the presence of RAVs needs to be confirmed before starting the treatment and setting the treatment direction. If RAVs vary depending on the regions and countries in which patients live, DAAs’ performance can be expected to differ accordingly. In the previous era of IFN-based treatment, IL28B genetic polymorphisms showed differences between Asian and non-Asian countries, and that treatment performance varied accordingly. [19–22] About 90% of the population of Korea are CC homozygotes, [2, 23, 24] and response of IFN-based treatment was relatively higher than in Western people. Because IL28B test is not routinely performed before treatment of CHC as guidelines suggested, [16] IL28B was not evaluated in these patients. Therefore, we could not figure out whether the presence of baseline RAVs differed significantly with respect to IL28B genotype.

Currently availabilities of DAAs differ from country to country. DCV + ASV began to be used in Japan after its approval in 2014, and it has been used in Korea since August 2015 after its insurance application was approved. Therefore, because of its limited use thus far, insufficient investigation has been made into the treatment performance of DCV + ASV and its related NS5A mutations. In this study, the real-life prevalence of NS5A RAVs detected at baseline in 142 Korean patients was investigated and was confirmed to be slightly higher prevalence than was previously reported. [25–27] According to data published in Japan in 2015, the incidence of NS5A mutations in Japan was 18.4%, which was higher than its incidence in non-Asian countries. [6] Although further studies are needed in the future, in regards to patients with CHC genotype 1b in Asia, frequent NS5A RAVs detected at baseline are considered a substantial limitation in DCV + ASV use, and other DAA regimens need to be considered because impact of RAVs is regimen specific. [12, 13]

According to our results, the RAV-positive incidence was not different in patients who was naïve or who failed prior IFN-based treatment. Furthermore, in patients with accompanying cirrhosis, the RAV-positive incidence was not statistically different. It is assumed that these patients have been infected with viruses for a long time and have retained viruses with diverse mutations, but again, the exact causes are not known. However, good treatment performance was observed in RAV-negative patients in this study, regardless of presence of cirrhosis or whether prior IFN-based treatments had failed. Patients who reached virologic response during treatment also reached SVR12 without much difficulty.

The number of patients with NS5A RAVs is slightly higher than the previously reported data of 12–18.4%. [25–27] Although further studies are needed in the future, according to our study results, the number of HCV genotype 1b–infected Korean patients without NS5A RAVs, and therefore suitable for treatment with DCV + ASV, might be slightly less than that in other regions or countries where the prevalence of these NS5A RAVs are less.

DAA-based treatment has relatively fewer adverse effects than IFN-based treatment. However, in the case with DCV, patients can have headaches, general weakness, and fatigue. Bradycardia arrhythmias have been observed in patients receiving amiodarone with DCV and sofosbuvir, with or without other drugs that lower heart rate. The mechanism for the bradycardia effect has not been clearly established; recent in vivo studies have demonstrated a sofobuvir amiodarone drug-drug interaction (DDI)-dependent selective nodal dysfunction resulting in bradycardia. [28] Moreover, ASV can have adverse effects including diarrhea, headache, hyperbilirubinemia, and increased transaminase level. Additionally, cases that were assumed to be immunoallergic hepatitis after DCV + ASV combination therapy have been reported. [29] According to a large scaled report of DCV + ASV therapy, 2.9% of patients discontinued the therapy as a result of ALT elevation. ALT elevation more than 1.25-fold the upper limit of normal was noted in 37.6% of patients. [30] Another large scaled study revealed that discontinuation due to liver injury occurred in 28 of 924 patients (3.0%). Other minor cause of discontinuation was fatigue, diarrhea, nausea, appetite loss, pneumonia, rash, platelet decrease and encephalopathy. [31] In a multi-center study among hemodialysis patients, 4 of 123 patients discontinued DCV + ASV therapy because of elevated serum alanine transaminase levels (*n* = 2), rash (*n* = 1), and HCC; all of these achieved SVR12. [32] In this study, three patients stopped treatment because of adverse effects, one of which was marked thrombocytopenia. One patient’s platelet count dropped from normal to very low 2 weeks after beginning treatment. This patient stopped the treatment, and the platelet levels returned to normal after steroid treatment. However, since the medicine can be accompanied by additional complications that can be life-threatening, hematologic disorders need to be carefully monitored during the treatment, though this patient has a history of cured ITP. Another patient had to stop treatment because of weakness and fatigue. Fatigue is often observed as an adverse effect of DCV + ASV therapy, and typically, it is not severe enough to stop treatment. However, the drug can result in general weakness and fatigue too severe for this patients to endure. The other patient discontinued DCV + ASV therapy due to elevated transaminases at week 7 after achieving virologic response at week 4. In this study, since adverse effects occurred within 2 month of the treatment in all cases, more careful observation is needed in the early period of treatment.

Limitations of the study are the limited sample size and dataset from a single center study. Although hepatitis B is endemic in Korea, Hepatitis C is not as prevalent as in Japan or in western countries. Thus, sample size is relatively small comparing other reports form these regions. These clinical data are collected in one tertiary referral hospital. Therefore, patients nationwide are included even though these results can not accurately reflect the patient across the country.

## Conclusions

In patients with HCV infection, when RAV is not present, DCV + ASV therapy has shown good treatment performance regardless of the presence of cirrhosis or previous IFN-based treatment experience. Therefore, DCV + ASV therapy can be expected to be actively used to treat CHC genotype 1b without NS5A RAVs. However, since there are a considerable number of RAV-positive cases, other DAA regimens need to be considered. The possibility of complications resulting from the treatment, although rare, needs to be considered and monitored with regular hepatic function tests.

## Abbreviations

ALT: alanine aminotransferase
AST: aspartate aminotransferase
ASV: asunaprevir
CHC: chronic hepatitis C
DAAs: direct-acting antivirals
DCV: daclatasvir
DM: diabetes mellitus
HTN: hypertension
IFN: Interferon
INR: international normalized ratio
ITP: idiopathic thrombocytopenic purpura
LC: liver cirrhosis
RAV: resistance-associated variant
SVR: sustained virologic response

## References

[1] KASL clinical practice guidelines: management of hepatitis C. Clin Mol Hepatol 2014, 20**:**89–136. 10.3350/cmh.2014.20.2.89 PMC4099340 25032178

[2] Lyoo K, Song MJ, Hur W, Hong SW, Kim CW, Bae SH, Choi JY, Choi SW, Shin E, Yoon SK (2011). Polymorphism near the IL28B gene in Korean hepatitis C virus-infected patients treated with peg-interferon plus ribavirin. J Clin Virol.

[3] Sulkowski MS, Gardiner DF, Rodriguez-Torres M, Reddy KR, Hassanein T, Jacobson I, Lawitz E, Lok AS, Hinestrosa F, Thuluvath PJ (2014). Daclatasvir plus sofosbuvir for previously treated or untreated chronic HCV infection. N Engl J Med.

[4] Jacobson IM, Dore GJ, Foster GR, Fried MW, Radu M, Rafalsky VV, Moroz L, Craxi A, Peeters M, Lenz O (2014). Simeprevir with pegylated interferon alfa 2a plus ribavirin in treatment-naive patients with chronic hepatitis C virus genotype 1 infection (QUEST-1): a phase 3, randomised, double-blind, placebo-controlled trial. Lancet.

[5] Furusyo N, Ogawa E, Nakamuta M, Kajiwara E, Nomura H, Dohmen K, Takahashi K, Satoh T, Azuma K, Kawano A (2013). Telaprevir can be successfully and safely used to treat older patients with genotype 1b chronic hepatitis C. J Hepatol.

[6] McPhee F, Suzuki Y, Toyota J, Karino Y, Chayama K, Kawakami Y, Yu ML, Ahn SH, Ishikawa H, Bhore R (2015). High sustained Virologic response to Daclatasvir plus Asunaprevir in elderly and cirrhotic patients with hepatitis C virus genotype 1b without baseline NS5A polymorphisms. Adv Ther.

[7] Poordad F, Dieterich D (2012). Treating hepatitis C: current standard of care and emerging direct-acting antiviral agents. J Viral Hepat.

[8] Manns M, Pol S, Jacobson IM, Marcellin P, Gordon SC, Peng CY, Chang TT, Everson GT, Heo J, Gerken G (2014). All-oral daclatasvir plus asunaprevir for hepatitis C virus genotype 1b: a multinational, phase 3, multicohort study. Lancet.

[9] Kumada H, Suzuki Y, Ikeda K, Toyota J, Karino Y, Chayama K, Kawakami Y, Ido A, Yamamoto K, Takaguchi K (2014). Daclatasvir plus asunaprevir for chronic HCV genotype 1b infection. Hepatology.

[10] Kao JH, Lee YJ, Heo J, Ahn SH, Lim YS, Peng CY, Chang TT, Torbeyns A, Hughes E, Bhore R, Noviello S. All-oral daclatasvir plus asunaprevir for chronic HCV genotype 1b infection: a sub-analysis in Asian patients from the HALLMARK DUAL study. Liver Int. 2016;36:1433-41. 10.1111/liv.13128 27009831

[11] Wei L, Zhang M, Xu M, Chuang WL, Lu W, Xie W, Jia Z, Gong G, Li Y, Bae SH, et al. A phase 3, open-label study of daclatasvir plus asunaprevir in Asian patients with chronic hepatitis C virus genotype 1b infection who are ineligible for or intolerant to interferon alfa therapies with or without ribavirin. J Gastroenterol Hepatol. 2016;31:1860-7. 10.1111/jgh.13379 27003037

[12] Sulkowski MS, Jacobson IM, Nelson DR (2014). Daclatasvir plus sofosbuvir for HCV infection. N Engl J Med.

[13] Wyles DL, Ruane PJ, Sulkowski MS, Dieterich D, Luetkemeyer A, Morgan TR, Sherman KE, Dretler R, Fishbein D, Gathe JC (2015). Daclatasvir plus Sofosbuvir for HCV in patients Coinfected with HIV-1. N Engl J Med.

[14] Hernandez D, Yu F, Huang X, Kirov S, Pant S, McPhee F. Impact of pre-existing NS5A-L31 or -Y93H minor variants on response rates in patients infected with HCV genotype-1b treated with Daclatasvir/Asunaprevir. Adv Ther. 2016;33:1169-79. 10.1007/s12325-016-0354-1 27287851

[15] Itakura J, Kurosaki M, Higuchi M, Takada H, Nakakuki N, Itakura Y, Tamaki N, Yasui Y, Suzuki S, Tsuchiya K (2015). Resistance-associated NS5A variants of hepatitis C virus are susceptible to interferon-based therapy. PLoS One.

[16] Korean Association for the Study of the L (2016). KASL clinical practice guidelines: management of hepatitis C. Clin Mol Hepatol.

[17] Chevaliez S (2011). Antiviral activity of the new DAAs for the treatment of hepatitis C virus infection: virology and resistance. Clin Res Hepatol Gastroenterol.

[18] Aghemo A, Colombo M (2013). Selection of resistant-associated variants to the NS5A inhibitor daclatasvir: revenge of the hepatitis C virus. Gastroenterology.

[19] Rangnekar AS, Fontana RJ (2012). Meta-analysis: IL-28B genotype and sustained viral clearance in HCV genotype 1 patients. Aliment Pharmacol Ther.

[20] Liu CH, Liu CJ, Lin CL, Liang CC, Hsu SJ, Yang SS, Hsu CS, Tseng TC, Wang CC, Lai MY (2008). Pegylated interferon-alpha-2a plus ribavirin for treatment-naive Asian patients with hepatitis C virus genotype 1 infection: a multicenter, randomized controlled trial. Clin Infect Dis.

[21] Liu CH, Liang CC, Liu CJ, Tseng TC, Lin CL, Yang SS, Su TH, Hsu SJ, Lin JW, Chen JH (2012). Interleukin 28B genetic polymorphisms and viral factors help identify HCV genotype-1 patients who benefit from 24-week pegylated interferon plus ribavirin therapy. Antivir Ther.

[22] Cavalcante LN, Abe-Sandes K, Angelo AL, Machado TM, Lemaire DC, Mendes CM, Pinho JR, Malta F, Lyra LG, Lyra AC (2012). IL28B polymorphisms are markers of therapy response and are influenced by genetic ancestry in chronic hepatitis C patients from an admixed population. Liver Int.

[23] Jeong SH, Jung YK, Yang JW, Park SJ, Kim JW, Kwon OS, Kim YS, Choi DJ, Kim JH (2012). Efficacy of peginterferon and ribavirin is associated with the IL28B gene in Korean patients with chronic hepatitis C. Clin Mol Hepatol.

[24] Jung YK, Kim JH, Ahn SM, Yang JW, Park SJ, Kim JW, Yeon JE, Kwon OS, Kim YS, Choi DJ (2013). Role of interleukin 28B-related gene polymorphisms in chronic hepatitis C and the response to antiviral therapy in Koreans. J Clin Gastroenterol.

[25] Paolucci S, Fiorina L, Mariani B, Gulminetti R, Novati S, Barbarini G, Bruno R, Baldanti F (2013). Naturally occurring resistance mutations to inhibitors of HCV NS5A region and NS5B polymerase in DAA treatment-naive patients. Virol J.

[26] Aissa Larousse J, Trimoulet P, Recordon Pinson P, Tauzin B, Azzouz MM, Ben Mami N, Cheikh I, Triki H, Fleury H (2015). Prevalence of hepatitis C virus (HCV) variants resistant to NS5A inhibitors in naive patients infected with HCV genotype 1 in Tunisia. Virol J.

[27] Nakamoto S, Kanda T, Wu S, Shirasawa H, Yokosuka O (2014). Hepatitis C virus NS5A inhibitors and drug resistance mutations. World J Gastroenterol.

[28] Regan CP, Morissette P, Regan HK, Travis JJ, Gerenser P, Wen J, Fitzgerald K, Gruver S, DeGeorge JJ, Sannajust FJ (2016). Assessment of the clinical cardiac drug-drug interaction associated with the combination of hepatitis C virus nucleotide inhibitors and amiodarone in guinea pigs and rhesus monkeys. Hepatology.

[29] Fujii Y, Uchida Y, Mochida S (2015). Drug-induced immunoallergic hepatitis during combination therapy with daclatasvir and asunaprevir. Hepatology.

[30] Sezaki H, Suzuki F, Hosaka T, Akuta N, Fujiyama S, Kawamura Y, Kobayashi M, Suzuki Y, Saitoh S, Arase Y, et al. The efficacy and safety of dual oral therapy with daclatasvir and asunaprevir for genotype 1b in Japanese real-life settings. Liver Int. 2017. doi:10.1111/liv.13384. 10.1111/liv.13384 28178397

[31] Ishigami M, Hayashi K, Honda T, Kuzuya T, Ishizu Y, Ishikawa T, Nakano I, Urano F, Kumada T, Yoshioka K, et al. Daclatasvir and asunaprevir treatment in patients with severe liver fibrosis by HCV genotype 1b infection: real world data. J Gastroenterol Hepatol. 2017. doi:10.1111/jgh.13779. 10.1111/jgh.13779 28258705

[32] Suda G, Furusyo N, Toyoda H, Kawakami Y, Ikeda H, Suzuki M, Arataki K, Mori N, Tsuji K, Katamura Y, et al. Daclatasvir and asunaprevir in hemodialysis patients with hepatitis C virus infection: a nationwide retrospective study in Japan. J Gastroenterol. 2017. doi:10.1007/s00535-017-1353-y. 10.1007/s00535-017-1353-y 28560477

